# UniSePT: an engineered broadly applicable Unique Secretory signal PepTide for high-yield biotherapeutic production in mammalian cell-based expression systems

**DOI:** 10.1186/s13036-026-00704-2

**Published:** 2026-06-08

**Authors:** Goutam Ulgekar, Satarupa Dutta, Ealisha Jain, Meenakshi Mansukhani, Harshada Thawari, Subeer S. Majumdar, Nirmalya Ganguli

**Affiliations:** 1 Gene and Protein Engineering Laboratory, National Institute of Animal Biotechnology, Hyderabad, Telangana India; 2 Gujarat Biotechnology University, Gandhinagar, Gujarat India

## Abstract

**Background:**

The yield of therapeutic proteins in mammalian cell-based production systems is often limited by inefficient secretion and reduced bioactivity caused by incompatible secretory signal peptides (SPs). Therefore, a broadly applicable signal peptide that can be fused to different proteins of interest to enhance their secretion and overall production is needed.

**Results:**

To address this challenge, we engineered a robust, broadly applicable Unique Secretory signal PepTide (UniSePT) by combining SP sequences from β-casein (CSN2) and β-lactoglobulin (BLG) proteins of Indian river buffalo. UniSePT demonstrated higher secretion efficiency than commonly used SPs, such as human serum albumin and preproinsulin SP. Importantly, this improved secretion does not compromise the bioactivity of the therapeutic proteins produced. Additionally, UniSePT showed minimal cleavage variability, supporting its compatibility with various therapeutic proteins.

**Conclusion:**

The newly developed UniSePT enhances the titer and bioactivity of therapeutic proteins in mammalian expression systems. Its broad applicability and improved secretion efficiency make it a promising tool for industrial-scale production, potentially reducing the cost of therapeutic protein production.

**Supplementary Information:**

The online version contains supplementary material available at 10.1186/s13036-026-00704-2.

## Background

The demand for recombinant biologics of mammalian origin has grown substantially in the post-genomic era, driven by their expanding applications in the treatment of various physiological conditions and diseases [1, 2]. Among these, therapeutic proteins constitute a significant class of biologics used to replace defective proteins, neutralize pathogenic molecules, and stimulate immune responses against diseases. Recombinant therapeutic proteins can be produced using various expression systems, including bacterial [3, 4], mammalian cell-based [5–7], and transgenic animal-based bioreactor platforms [8, 9]. Although bacterial systems are simple and cost-effective, they frequently fail to produce bioactive mammalian proteins because they lack the cellular machinery required for complex post-translational modifications (PTMs), such as glycosylation and disulfide bond formation [10]. Consequently, mammalian expression systems, including Chinese Hamster Ovary (CHO) and Human Embryonic Kidney 293 (HEK293) cell lines, are preferred for producing complex therapeutic proteins because of the presence of cellular organelles, such as the endoplasmic reticulum (ER) and Golgi apparatus, which support advanced post-translational modification machinery [11–13]. However, several therapeutic proteins expressed in mammalian cells or transgenic animals still exhibit suboptimal yields or compromised bioactivity due to improper PTMs, thereby increasing production costs and impeding scalability [14]. One of the major limiting factors is the use of inefficient or incompatible signal peptides (SPs), which fail to direct nascent polypeptides efficiently into the ER for proper folding, PTMs, and secretion [15].

Signal peptides (SPs) are short N-terminal sequences, typically 15–25 amino acids in length, that direct nascent polypeptides to the endoplasmic reticulum (ER) for co-translational translocation [16, 17]. Although numerous native and synthetic SPs have been explored to enhance recombinant protein secretion, their effectiveness is often highly dependent on the specific protein cargo and the expression system employed [15]. Commonly used SPs, such as those derived from human serum albumin [18] and pre-proinsulin [19], are widely adopted in both academic and industrial settings. However, they frequently fail to consistently enhance the secretion of diverse therapeutic proteins across mammalian cell-based production platforms [15]. A recent study developed a high-throughput computational pipeline to design optimized signal peptides, significantly enhancing recombinant protein secretion in CHO cells and revealing key determinants, such as hydrophobicity patterns and mRNA stability [20]. However, a ready-made, broadly applicable signal peptide that can be used instantly with a wide variety of protein cargoes remains a challenge.

These limitations primarily arise from suboptimal signal peptide architecture, particularly the lack of a sufficiently hydrophobic core and/or an efficiently recognized signal peptidase cleavage site [16, 21]. Some studies have explored the utility of SP from milk proteins, like αs1-casein, for enhancing recombinant protein secretion in mammalian cells [22–24]. The CSN2 signal peptide has been extensively reported to confer highly efficient ER targeting [25–27]; however, its cleavage site can be incompatible with certain heterologous proteins, potentially leading to retention or sequestration of the mature protein [28]. In contrast, the BLG signal peptide has been highlighted for its precise and reliable signal peptidase cleavage, enabling accurate N-terminal processing and proper maturation of recombinant proteins [29]. However, a comparative study evaluating the secretion efficiency of signal peptides from various milk proteins is lacking.

In this study, for the first time, we aimed to compare the signal peptides of various milk protein genes and subsequently develop and validate a broadly applicable signal peptide to enhance secretion efficiency, thereby increasing the yield while maintaining proper bioactivity of recombinant therapeutic proteins in mammalian expression systems.

We first assessed the secretion potential of SPs of different milk proteins of Indian river buffalo (*Bubalus bubalis*) origin by tagging them with recombinant proteins. Based on previous studies and our in silico analysis, we selected two well-characterized signal peptides derived from milk proteins, β-casein (CSN2) and β-lactoglobulin (BLG). The analysis revealed that their functional attributes complemented each other. We hypothesized that a rationally engineered signal peptide combining the robust ER-targeting efficiency of CSN2 with the precise cleavage properties of BLG could yield a superior signal peptide. To test our hypothesis, we chose two recombinant proteins, human interferon gamma (hIFNγ) and enhanced green fluorescent protein (EGFP), based on their N-terminal amino acid sequences and their known roles in signal peptide cleavage efficiency. This approach led to the generation of a novel signal peptide, UniSePT, which enabled the enhanced production of recombinant proteins with retained bioactivity, outperforming the currently available industrially relevant signal peptides. Our findings have broad implications for the production of cost-effective, high-yield biologics and contribute to ongoing efforts to optimize vector design in mammalian cell-based biotechnology applications.

## Methods

### Retrieving and annotation of secretory signal peptide sequences of milk proteins of buffalo

The amino acid sequences of six predominant milk proteins (*csn1s1*,* csn1s2*,* csn2*,* csn3*,* lalba*,* and blg*) were retrieved from UniProt (https://www.uniprot.org/). Signal peptide sequences were determined using the UniProt and SignalP 6.0 tool (https://services.healthtech.dtu.dk/services/SignalP-6.0/) based on a deep convolutional and recurrent neural network architecture, including a conditional random field [30].

### Identification of sub-cellular localization, secretion efficiency, and cleavage discrepancy of SPs

The subcellular localization of human interferon gamma (hIFNγ) tagged with different milk protein SP was investigated using DeepLoc 1.0 (http://www.cbs.dtu.dk/services/DeepLoc/). For eukaryotic proteins, eight different locations were predicted: nucleus, cytoplasm, Extracellular, Cell membrane, Endoplasmic Reticulum, Golgi apparatus, lysosome/vacuole, and peroxisome. The N, H, and C regions, as well as the regions to be designated as SP for all milk proteins, were predicted using the Phobius tool (https://www.ebi.ac.uk/Tools/pfa/phobius/). The hydropathic index of the designated milk protein SP was determined using the GRAVY calculator tool (https://www.gravy-calculator.de/). The efficiency and cleavage-site discrepancies of these SPs, when tagged with hIFNγ and EGFP, respectively, were determined using PrediSi (http://www.predisi.de/) and SignalP 6.0. The amino acid sequence of therapeutic proteins were retrieved from THPdb, an online database [31].

### Cloning and generation of the expression constructs

Different milk SPs (CSN1S1, CSN1S2, CSN2, CSN3, LALBA, and BLG), UniSePT SP, or industrially relevant SPs (hALB and hINS) were fused with hIFNγ or EGFP cDNA by overhang PCR using the parent vector pCMV-hIFNγ-IRES2-EGFP [32]. The forward primers contained an SP sequence in overhang. PCR was performed using a 10ng pCMV-hIFNγ-IRES2-EGFP plasmid template, and the desired amplicon was excised from the agarose gel. The excised amplicon was eluted using the HiYield Plus™ Gel/PCR DNA Mini Kit (RBC, #QDF100) according to the manufacturer’s protocol. The eluted DNA was then subjected to T4 Polynucleotide Kinase (NEB, #M0201L) treatment for phosphorylation at the 5’ends. The phosphorylated DNA was then ligated using T4 DNA Ligase (NEB, #M0202L), and the ligated product was transformed into the DH5α strain of ultracompetent E. coli cells and plated on agar plates supplemented with kanamycin (Sigma-Aldrich, #60615-25G) at a working concentration of 30 µg/mL. Plasmids were isolated from colonies obtained after overnight growth in LB medium, and the constructs were confirmed by Sanger sequencing.

### Cell lines

HEK293T cells were revived in DMEM high glucose (HiMedia, #AT066-1 L) containing 10% FBS and 1X Antibiotic Solution (HiMedia, #A002). After achieving 80% confluency, the cells were trypsinized using 0.05% trypsin-EDTA (HiMedia, #TCL033) and split according to experimental needs. CHO K1 cells were revived in DMEM/Ham’s F12 (1:1) containing 10% FBS and 1X Antibiotic Solution. The cells were trypsinized and split according to experimental needs, like HEK293T cells. For transient transfection, cells were seeded in appropriate culture plates and allowed to reach 70–80% confluence. Plasmid DNA encoding the desired cassette (supplementary figure S1B, and S3B) was transfected using FA6-bPEI as described previously [32]. Briefly, appropriate amount of DNA was dissolved in Dulbecco phosphate buffered saline (sigma-aldrich, USA). After 5 min of incubation, FA6-bPEI was added to the solution at 1:1 ratio. The solution was incubated for 8 min to allow complex formation before being added to the cells. After 4 h, the transfection medium was replaced with fresh complete medium. The cells were further incubated for 24–48 h prior to downstream analysis. All experiments were performed using cells at passage 2.

### Isolation and culture of mammary epithelial cells from the goat mammary gland

Mammary gland tissue was collected from a local abattoir in 1× PBS supplemented with 1X Antibiotic Solution. Clean parenchymatous tissue was excised, finely minced, and subjected to enzymatic digestion in DMEM/Ham’s F12 (1:1) containing 2% FBS, collagenase, and hyaluronidase (400 U/mL each) and 1X Antibiotic Solution. Digestion was performed at 37 °C in a shaking water bath (150 rpm), and the dissociated cells were collected after 60 and 120-min. Cell suspensions were filtered through a 40 μm strainer, centrifuged at 2000 rpm for 10 min at 4 °C, and washed twice with ice-cold HBSS containing 2% FBS, the above enzymes, and 1X Antibiotic Solution. The pellet was treated with 0.05% trypsin–EDTA for 5 min at 37 °C, centrifuged, and resuspended in mammary epithelial cell growth (MECG) medium consisting of DMEM: Ham’s F12 (1:1) supplemented with 10% FBS, insulin (10 µg/mL), transferrin (5.5 µg/mL), sodium selenite (5 ng/mL), epidermal growth factor (10 ng/mL), and 1X Antibiotic Solution. Cells were seeded in T-25 flasks and cultured at 37 °C in a CO₂ incubator for 24–48 h. Differential trypsinization was performed to remove the fibroblasts.

### Flow cytometry

Flow cytometric analysis was performed using a BD FACSMelody Cell Sorter (RRID: SCR_023209) to compare the transfection efficiency of constructs with different signal peptides. The transfected cells (HEK293T, CHO K1 and GMEC) were trypsinized after 36 h of transfection to generate a single-cell suspension. The single-cell suspension was analyzed to determine the percentage of transfected cells through quantification of the EGFP expression in the cells. An excitation wavelength of 488 nm was used in flow cytometry to detect the EGFP reporter protein in transfected cells. Untransfected cells were used to gate single cells and the EGFP-negative population. The percentage of transfected cells was quantified using FlowJo (RRID: SCR_008520).

### qPCR analysis of plasmid copy number

Genomic DNA was isolated from transfected CHO-K1 cells using DNeasy Blood and Tissue Kits for DNA Isolation(Qiagen, #69504). Plasmid copy numbers were determined using qPCR in the Bio-Rad CFX96 Real-Time PCR Detection System (RRID: SCR_018064). A standard curve was plotted using known copies of plasmid DNA spiked into the genomic DNA of wild-type CHO K1 cells. Copy numbers were determined from 20ng of genomic DNA from transfected CHO K1 cells by interpolating the values from the standard curve. The 10 µL qPCR reaction mix consisted of 1 µL template DNA (20 ng/µL), 5 µL SYBR Green master mix (Thermo Scientific #4367659), 0.5 µL of each forward and reverse oligonucleotide (10 µM each) of human interferon gamma (FP: 5′- TGACCAGAGCATCCAAAAGA − 3′; RP: 5′-CTCTTCGACCTCGAAACAGC − 3′) as well as mouse beta actin (FP: 5′- GCAAAGACCTGTACGCCAAC-3′; RP: 5′-GATCCACACGGAGTACTTGC-3′), and 3 µL of nuclease-free water was subjected to heating at 95 °C for 10 min, followed by 40 cycles of 95 °C for 10 s, 50 °C for 30 s, and elongation for 30 s at 72 °C, then 60 °C for 30 min for data acquisition.

### High content screening (HCS) microscopy analysis

High content screening analysis was performed to quantify the ER localization of hIFNγ or EGFP tagged with different signal peptides using the Operetta CLS^™^ High-Content Analysis System (PerkinElmer, Waltham, MA, USA) (RRID: SCR_018810). Goat mammary epithelial cells were seeded in a 96-well phenoplate at a density of 1 × 10^4 cells/ml. Upon reaching 70% confluency, the cells were co-transfected with the plasmid constructs pCMV-EGFP or pCMV-hIFNγ-IRES2-EGFP, along with various signal peptides (CSN2, BLG, and UniSePT) and mCherry-ER-3 construct (Addgene #55041) expressing mCherry protein tagged with ER targeting signal sequence (calreticulin+ KDEL). Brefeldin A (Thermo Scientific #00-4506-51) treatment was given to transfected cells (18-hour post-transfection) for 8 h to inhibit ER to Golgi transport. Cells were fixed using 2% paraformaldehyde solution and nucleus was counter-stained using hoescht 33342. The Cells were then visualised using DAPI (nucleus), FITC (EGFP) and TRITC (mCherry) channels for the study based on EGFP. Whereas, cells were immune-stained with primary mouse anti-hIFNγ antibody(Santa Cruz Biotechnology, #sc-373727) and anti-mouse IgG antibody tagged with AlexaFlour 647 (Thermo Scientific, #A32728) for hIFNγ protein(Cy5 channel). Thermo scientific HCS studio software was used to evaluate the co-localization efficiency of mCherry with ER-localized EGFP and hIFNγ.

### Protein-protein interaction study

Protein-protein interaction studies were performed using ClusPro 2.0 software [33]. To this end, the PDB file (PDB ID:7QWQ) of the ternary complex of the ribosome nascent chain with Signal Recognition Particle 54 (SRP54) and Nascent Polypeptide-Associated Complex (NAC) was retrieved. Simultaneously, 3D models of the signal peptides (CSN2, BLG, and UniSePT) were generated using AlphaFold (https://alphafoldserver.com/). Ribosomal RNA associated with ternary complex was removed using the ChimeraX tool [34], while maintaining the integrity of the remaining protein structure. Protein-protein docking was performed using ClusPro 2.0, and clusters were visualized using PyMOL 3.1(https://www.pymol.org/) to evaluate interaction of UniSePT SP with methionine rich domain (M-domain) of SRP54.

### Quantification of EGFP and hIFNγ

Transfected cells were lysed using RIPA lysis buffer (Thermofisher scientific, #89900) followed by estimation of total protein. Fluorimetry was used to quantify EGFP using EnSpire^®^ Multimode Plate Reader (Perkin-Elmer, USA). Enzyme-linked immunosorbent assay (ELISA) was performed BD OptEIA Human IFN-γ ELISA Set (BD Bioscience #555142) according to the manufacturer’s instructions. Total protein was estimated and loaded into the wells of the microtiter plate at an initial concentration of 4800 pg/ml, with dilutions up to 18.75pg/ml. Absorbance was measured at 450 nm with λ correction at 570 nm using a BioTek ELISA plate reader (RRID: SCR_018261).

### Kynurenine assay

Kynurenine assay was performed by inducing Thp1 cells with human interferon gamma, followed by detection of kynurenine in the spent media using the Ehrlich reagent (HiMedia, #R005). To this end, Thp1 cells were seeded in a T-25 flask and reseeded into 12-well plates once the desired growth had been achieved. Various amounts (400–1600 U/ml) of commercially available recombinant hIFNγ (PeproTech, USA, #300-02) were added to Thp1 cells in different wells and incubated for 72 h. In the next step, 160 µl of spent media was mixed with 10 µl of trichloroacetic acid (TCA) in MCT and incubated at 50 °C for 30 min. The treated samples were centrifuged at 21,000 × g for 10 min, and 100 µL of the supernatant was transferred to a 96-well plate. Ehrlich’s reagent (100 µL) was added to the supernatant and incubated at room temperature for 10 min. Absorbance was measured at 490 nm using an ELISA plate reader. A standard curve was plotted with absorbance against the concentration of recombinant hIFNγ, which was used to interpolate the bioactivity of hIFNγ expressed in transfected cells.

### Statistical analysis

Statistical analyses were conducted using GraphPad Prism software version 8.0 (GraphPad Software Inc.). Data are represented as mean ± SEM from at least three independent experiments. For comparisons between the two groups, statistical significance was determined using a two-tailed Student’s t-test. For experiments involving comparisons between more than two groups, one-way analysis of variance (ANOVA) was performed, followed by Tukey’s multiple comparisons test to assess pairwise differences between groups. Statistical significance was set at *p* < 0.05. The specific statistical tests used for each experiment are indicated in the corresponding figure legends.

## Results

### In silico profiling of Indian river buffalo milk protein signal peptides identifies CSN2 for secretion strength and BLG for cleavage accuracy

**Fig. 1:**
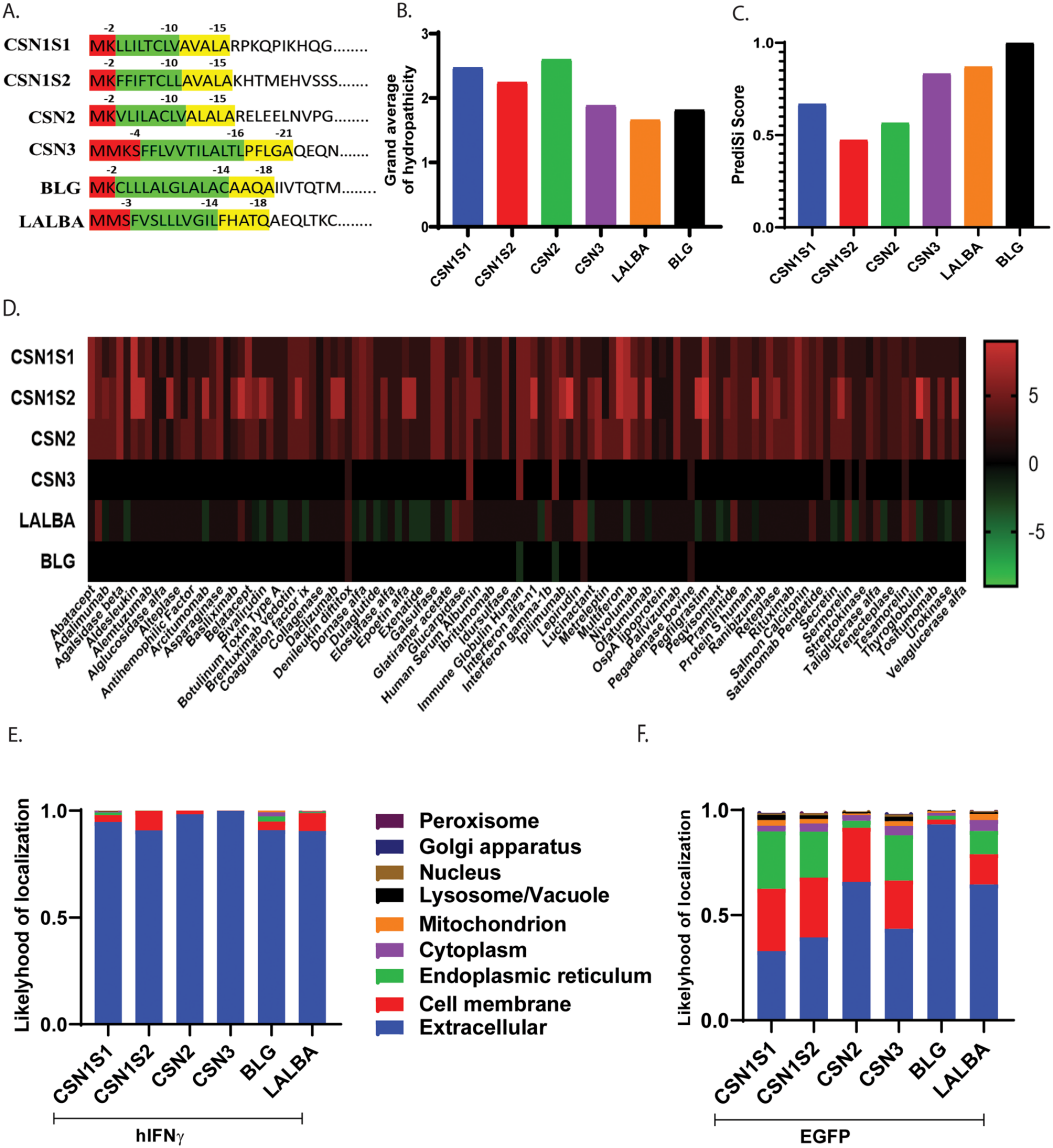
In Silico Profiling of Indian River Buffalo Milk Protein Signal Peptides Identifies CSN2 for Secretion Potency and BLG for Precise Cleavage Efficiency (**A**) Annotated and marked regions for various milk protein SPs using the Phobius tool showing N-region (red), H-region (green) and C-region (yellow). (**B**) Analysis of the grand average of the hydropathicity of milk protein signal peptides (Csn1s1, Csn1s2, Csn2, Csn3, Lalba, and Blg) using the ProtScale Tool. (**C**) Predicted cleavage efficiency of milk protein signal peptides (Csn1s1, Csn1s2, Csn2, Csn3, Lalba, and Blg) using the PredSi tool. (**D**) Prediction of cleavage discrepancy of milk protein signal peptides (Csn1s1, Csn1s2, Csn2, Csn3, Lalba, and Blg) with FDA-approved therapeutic proteins using the PredSi tool. (**E**) Predicted subcellular localization of milk protein signal peptides (Csn1s1, Csn1s2, Csn2, Csn3, Lalba, and Blg) with human interferon gamma using DeepLoc 1.0. (**F**) Predicted subcellular localization of milk protein signal peptides (Csn1s1, Csn1s2, Csn2, Csn3, Lalba, and Blg) with enhanced green fluorescent protein using DeepLoc 1.0

We selected proteins αS1-Casein (CSN1S1), αS2-Casein (CSN1S2), β-Casein (CSN2), Kappa Casein (CSN3), β-Lactoglobulin (BLG), and α-Lactalbumin (LALBA) of less explored Indian river buffalo for our study, as they are the major proteins present in buffalo milk. We annotated and marked the regions for SPs of various milk proteins using the Phobius tool (Fig. 1A). A hydropathic plot of all SPs was generated using the ProtScale tool, and cleavage efficiency was analyzed using the prediSi. Based on this analysis, *CSN2*-SP had the highest hydropathy index among all SPs, indicating its superiority for secretion (Fig. 1B), whereas BLG-SP exhibited the highest prediSi score for cleavage efficiency (Fig. 1C). Furthermore, a detailed in silico analysis revealed minimal cleavage discrepancies for BLG-SP when tagged with market-available major recombinant proteins compared to other milk protein SPs (Fig. 1D). A subcellular localization study using DeepLoc 1.0 predicted the secretion of human interferon gamma (hIFNγ), a well-known secreted therapeutic protein, with a score > 0.6 (Threshold = 0.58) when tagged with any of the milk protein SPs, followed by its native SP (Fig. 1E). Interestingly, a similar study with a widely used recombinant non-secretory protein, EGFP, predicted suboptimal secretion when tagged to CSN1S1, CSN1S2, and CSN3 SPs, whereas CSN2, BLG, and LALBA exhibited above-threshold scores, that is,>0.6 (Fig. 1F). However, among CSN2, BLG, and LALBA, BLG-SP outcompeted the other two SPs. Based on these in silico analyses, we inferred that among the chosen milk proteins, CSN2 SP exhibited the highest secretion efficiency, as indicated by its strong hydrophobic core, whereas BLG-SP exhibited the highest cleavage efficiency, as indicated by its favorable cleavage sites. Subcellular localization studies have shown that BLG-SP may outcompete other SPs for secretion efficiency when tagged to non-secretory proteins such as EGFP. Hence, we included only CSN2 and BLG SPs from the pool of milk protein SPs for further studies.

### Differential secretion and cleavage fidelity of CSN2 and BLG signal peptides is dependent on the recombinant protein cargo

**Fig. 2:**
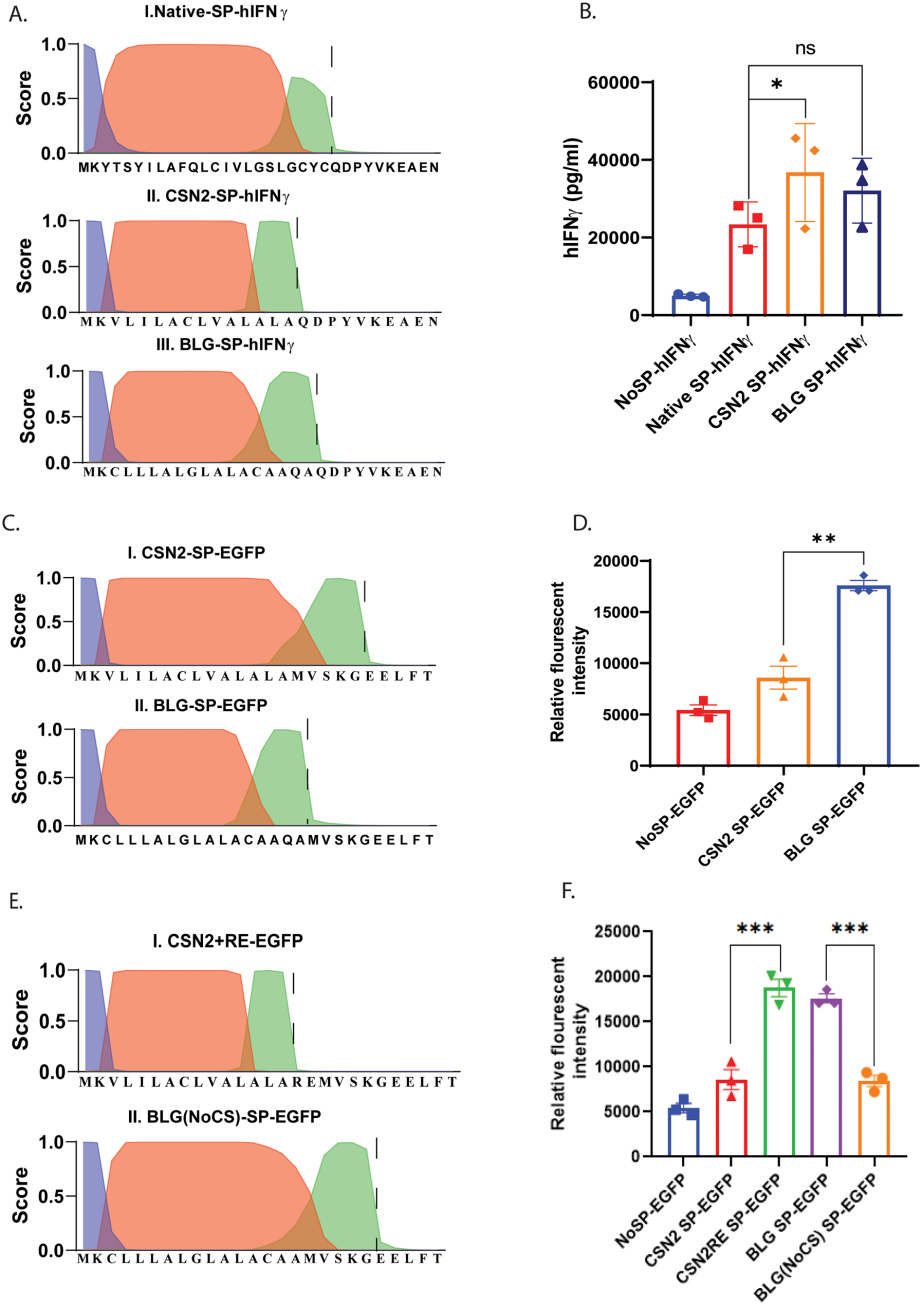
Recombinant cargo–dependent variation in the secretion efficiency and signal peptide cleavage of CSN2 and BLG signal sequences. (**A**) Cleavage prediction plot showing N-region (blue), H-region (orange) and C-region (green) of Native, CSN2 and BLG SP tagged with hIFNγ protein, with a black dashed line showing the cleavage site. (**B**) Quantification of hIFNγ in the spent media of CHO K1 cells transfected with plasmid constructs having human interferon gamma (hIFNγ) with CSN2 and BLG SP, (**C**) Cleavage prediction plot of CSN2 and BLG SP tagged with EGFP protein with dashed line showing cleavage site. (**D**) Quantification of EGFP in the spent media of CHO K1 cells transfected with plasmid constructs expressing EGFP tagged with CSN2 and BLG SP. (**E**) Cleavage prediction plot of CSN2 + RE and BLG-NoCS SP tagged with EGFP protein, with a dashed line showing the cleavage site. (**F**) Quantification of EGFP in the spent media of CHO K1 cells transfected with a plasmid construct expressing EGFP with CSN2 and BLG SP. The plasmid construct with no signal sequences tagged to the cDNA of hIFNγ/EGFP (NoSP-hIFNγ/EGFP) at the N-terminus was used as a control. The data indicate the mean and SEM, *n* = 3 independent experiments. Data were analyzed using a two-tailed t-test and one-way analysis of variance (ANOVA) followed by Tukey’s multiple comparisons test, ns (non-significant), *p ≤ 0.05, **p ≤ 0.01, ***p ≤ 0.001)

In silico analysis using SignalP 6.0 software predicted that CSN2 and BLG-SP exhibit cleavage efficiency similar to that of native SP, without any discrepancy in the case of hIFNγ protein (Fig. 2A). However, an in vitro study involving the secretion of hIFNγ protein from CHO K1 cells showed significantly higher secretion in the case of CSN2 SP than in the case of the native signal peptide (Fig. 2B and Fig. S1B). Interestingly, BLG-SP showed no significant increase in the secretion efficiency of hIFNγ compared to that of native SP. We confirmed these results in human embryonic kidney (HEK293T) and primary goat mammary epithelial cells (GMEC) as well (Fig. S2). A similar study with EGFP showed that CSN2-SP exhibits cleavage discrepancy (+ 5AA), whereas BLG-SP showed no discrepancy based on in silico analysis (Fig. 2C and Fig. S3B). We also found that CSN2-SP could not efficiently secrete EGFP based on flourimetric analysis of spent media in CHO K1 cells. However, BLG-SP successfully secreted EGFP, consistent with in silico predictions (Fig. 2D). Transfection efficiency was analyzed by flow cytometry for EGFP expression, which showed no significant change across all experiments (Fig. S4).

### Modified CSN2 and BLG-SP confirms the role of c-region integrity in protein secretion

We modified CSN2-SP by adding two more amino acids, arginine (R) and glutamine (E), from the native CSN2 protein (CSN2 + RE). Furthermore, we tagged the BLG signal peptide without the c-region with the N-terminus of EGFP (BLG-NoCS). In-silico analysis predicted the inclusion of arginine (R) and glutamate (E) with EGFP in the case of CSN2 + RE SP and the inclusion of five amino acids of EGFP in the case of BLG-NoCS SP (Fig. 2E). Fluorimetric analysis of spent media revealed that CSN2 + RE-SP efficiently secreted EGFP, suggesting that cleavage-site rescue occurs with the inclusion of additional amino acids. Moreover, BLG-NoCS SP showed negligible EGFP levels in the spent media (Fig. 2F), indicating a loss of cleavage efficiency, as predicted by our in silico analysis. Transfection efficiency was measured by copy number analysis, which showed no significant changes in any of the experiments (Fig. S5-S7).

### Decoupling hydrophobicity and cleavage determinants through hybrid signal peptide engineering enhances protein secretion

**Fig. 3:**
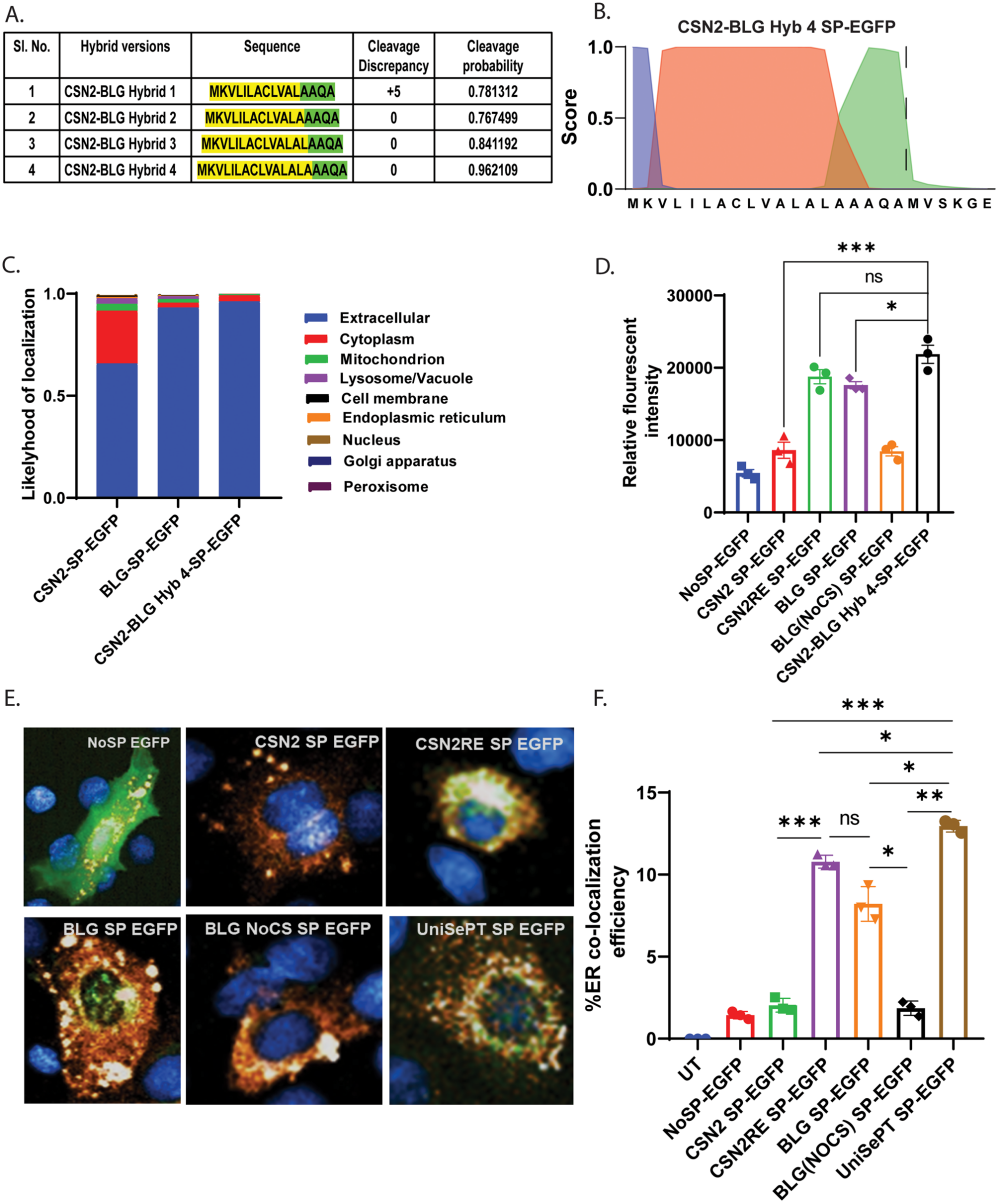
Rational hybrid signal peptide design uncouples hydrophobic core strength and cleavage precision to enhance recombinant protein secretion. (**A**)Table showing cleavage discrepancy and probability of hybrid versions of CSN2 and BLG SP (**B**) Cleavage prediction plot showing N-region (blue), H-region (orange) and C-region (green) of CSN2 BLG Hyb 4 SP with EGFP protein with a dashed line showing the cleavage site. (**C**) Prediction of localization of enhanced green fluorescent protein (EGFP) with CSN2, BLG, and CSN2 BLG Hyb 4 signal peptide using the DeepLoc 1.0 tool. (**D**) Quantification of EGFP in the spent media of CHO K1 cells transfected with the plasmid construct pCMV-NoSP-EGFP and different native and modified signal peptides (CSN2, CSN2 + RE, BLG, BLG(NoCS), and CSN2 BLG Hyb 4 signal peptide) 24 h post-transfection. (**E**) Immunocytochemical images of goat mammary epithelial cells transfected with plasmid constructs expressing EGFP without a signal peptide (NoSP-EGFP) and with different native and modified signal peptides (CSN2, CSN2 + RE, BLG, BLG(NoCS), and UniSePT signal peptides) 24 h post-transfection. (**F**)High-content screening of goat mammary epithelial cells transfected with different native and modified signal peptides (CSN2, CSN2 + RE, BLG, BLG(NoCS), and UniSePT signal peptide) 24 h post-transfection. A plasmid construct expressing EGFP without a signal peptide (NoSP-EGFP) was used as a control. The data indicates mean and SEM, *n* = 3 independent experiments. The data indicates mean and SEM, *n* = 3 independent experiments. Data were analyzed by one-way analysis of variance (ANOVA) followed by Tukey’s multiple comparisons test, ns (non-significant), *p ≤ 0.05, **p ≤ 0.01, and ***p ≤ 0.001)

Based on the outcomes of previous experiments, we hypothesized that a hybrid of CSN2 and BLG-SP could address existing issues with efficient secretion and cleavage compatibility with tagged proteins. We generated different combinations of hybrid signal peptides consisting of the intact hydrophobic core of CSN2 and the c-region of BLG-SP (CSN2-BLG Hyb 1–4). In silico analysis predicted the highest cleavage probability of EGFP when tagged with CSN2-BLG Hyb 4 without the inclusion of any additional amino acids (Fig. 3A and B). Furthermore, a subcellular localization study using DeepLoc 1.0 predicted the enhanced secretion of EGFP with CSN2-BLG Hyb 4 compared to its parental SPs, CSN2 and BLG-SP (Fig. 3C). Fluorimetric analysis of the spent media confirmed a significantly higher secretion efficiency in CSN2-BLG Hyb 4 than in CSN2 and BLG-SP (Fig. 3D). Considering the functional attributes, the newly developed CSN2-BLG Hybrid 4 was renamed UniSePT, that is, a broadly applicable Unique Secretory signal PepTide. To investigate the ability of UniSePT-SP to translocate the tagged protein into the ER, we co-transfected different SP-tagged EGFP constructs with ER localization signal-tagged mCherry constructs. Quantification of ER localization through high-content screening microscopy of co-transfected cells demonstrated that the inclusion of UniSePT-SP resulted in significantly higher ER co-localization of EGFP with mCherry compared to other signal peptides (Fig. 3E and F). This confirmed the more efficient translocation of the exogenous protein into the ER lumen with the help of UniSePT.

### UniSePT drives superior hIFNγ secretion and retains functional activity compared to native signal peptides

**Fig. 4:**
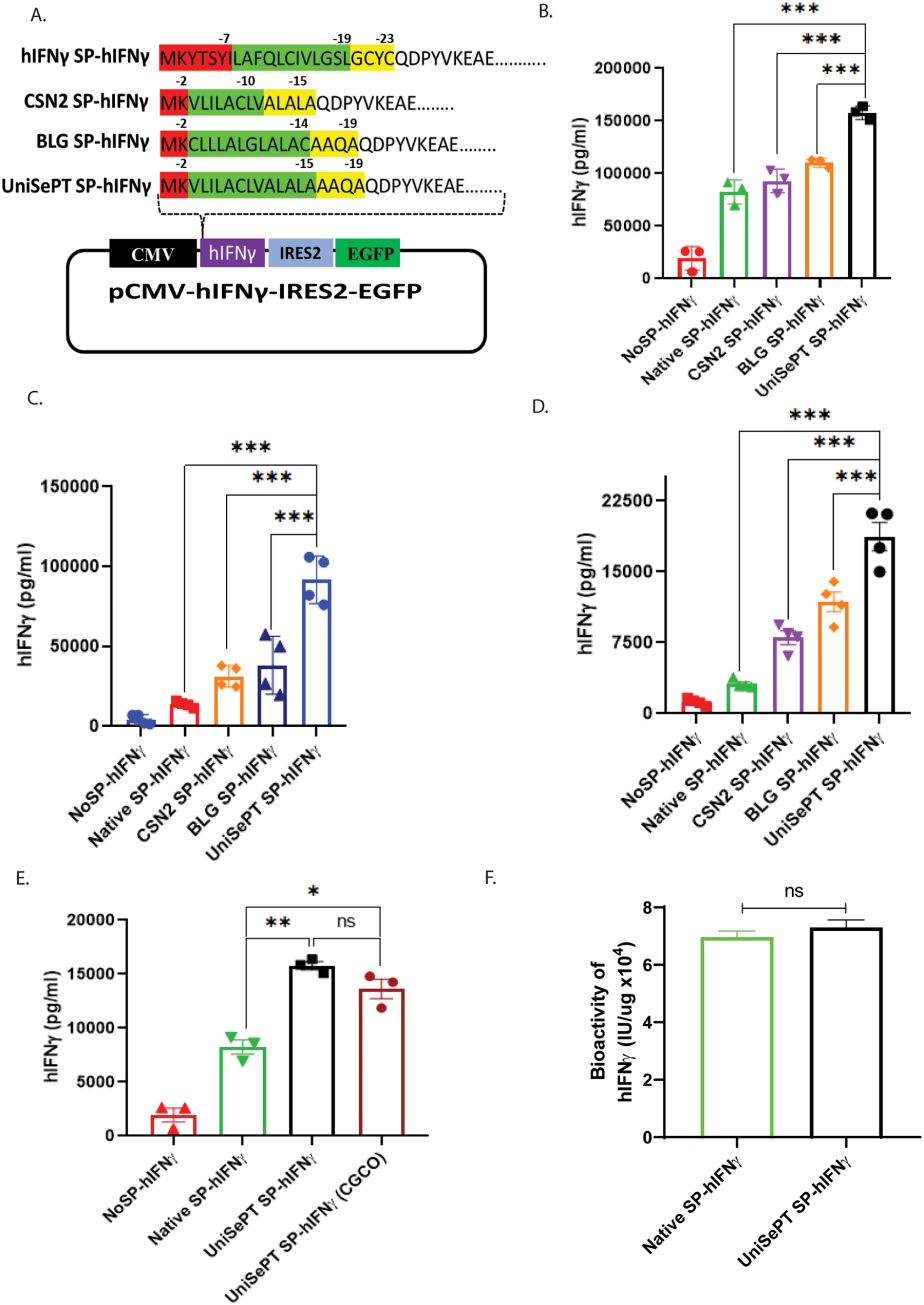
UniSePT enhances hIFNγ secretion efficiency and retains bioactivity compared to native signal peptides. (**A**) The plasmid construct showing signal sequences of native, CSN2, BLG, and UniSePT attached to the cDNA of hIFNγ at the N-terminal end. (**B-D**) Quantification of hIFNγ in spent media of HEK293T, CHO K1 and Goat mammary epithelial cells transfected with plasmid construct having human interferon gamma (hIFNγ) tagged with Native, CSN2, BLG and UniSePT SP respectively, (**E**) Quantification of hIFNγ in spent media of CHO K1 cells transfected with plasmid construct expressing hIFNγ tagged with original (buffalo origin) and codon optimised (Chinese hamster) UniSePT signal peptide (CGCO) (**F**) Bioactivity assay of hIFNγ tagged with native hIFNγ and UniSePT SP by kynurenine assay. The plasmid construct with no signal sequences attached to the cDNA of hIFNγ (NoSP-hIFNγ) at the N-terminal end was used as a control. The data indicate mean and SEM, *n* = 3 independent experiments. Data were analyzed by one-way analysis of variance (ANOVA) followed by Tukey’s multiple comparisons test, ns (non-significant), *p ≤ 0.05, **p ≤ 0.01, ***p ≤ 0.001)

To analyze the secretion efficiency of the newly developed UniSePT in guiding a recombinant therapeutic protein, we transfected HEK293T, CHO K1, and GMEC with a plasmid construct that expressed hIFNγ tagged with UniSePT (Fig. 4A). We also considered CSN2-SP and BLG-SP as the control groups. Quantification of hIFNγ expression in the spent media of transfected cells showed significantly higher secretion efficiency (> 1.5 fold) with UniSePT across different cell lines and primary cells compared to the native SP of hIFNγ (Fig. 4B-D). Furthermore, we tested the effect of codon optimization of UniSePT-SP on secretion efficiency. Surprisingly, we found that there were no significant changes in UniSePT with the original buffalo-derived codon sequence compared to the codon-optimized sequence based on a study performed on CHO K1 cells (Fig. 4E). To confirm that the hIFNγ expressed using UniSePT-SP retained bioactivity, we performed a kynurenine assay. These results showed that hIFNγ secreted through UniSePT retained bioactivity compared to hIFNγ produced using its native-SP. Upon interpolation with commercially available recombinant hIFNγ as a standard, the bioactivity of hIFNγ secreted using UniSePT was found to be ~ 7.3 × 10^3 IU/ml, as compared with native hIFNγ SP, which was at ~ 6.9 × 10^3 IU/ml (Fig. 4F).

### Superior performance of UniSePT over industrial signal peptides in hIFNγ secretion

**Fig. 5:**
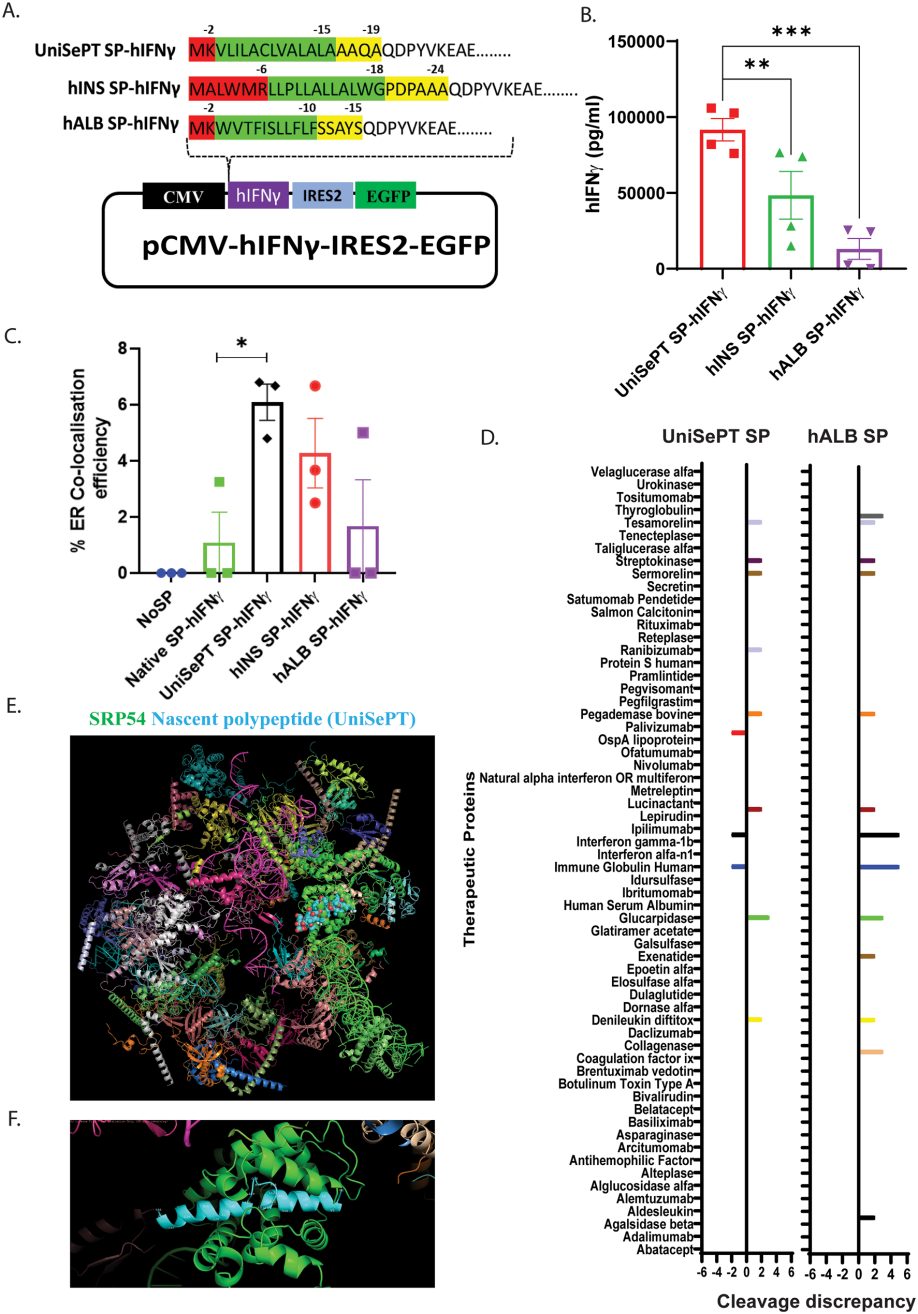
Enhanced hIFNγ secretion by UniSePT compared with established industrial signal peptides. (**A**) The plasmid construct showed signal sequences of UniSePT, human insulin (hINS), and human serum albumin (hALB) SP tagged to the cDNA of the hIFNγ at the N-terminal end. (**B**) Quantification of hIFNγ in the spent media of CHO K1 cells transfected with a plasmid construct expressing human interferon gamma (hIFNγ) tagged with UniSePT, human insulin (hINS), and human serum albumin (hALB). (**C**) High-content screening of goat mammary epithelial cells transfected with a plasmid construct expressing hIFNγ tagged with UniSePT, human insulin (hINS), and human serum albumin (hALB) SP (**D**) In silico study using major commercially important recombinant proteins with UniSePT and hALB SP to confirm the broad compatibility of the UniSePT SP with other therapeutic proteins. (**E**) Docking analysis of UniSePT signal peptide (turquoise) with SRP54 protein (green) using ClusPro 2. software shows the proper binding of UniSePT signal peptide with SRP54. (**F**) Image showing UniSePT signal peptide (turquoise color) binding to M-domain (methionine-rich domain) of SRP54 (green color). The data indicates mean and SEM, n= 3 independent experiments. Data were analyzed using a two-tailed t-test and one-way analysis of variance (ANOVA) followed by Tukey’s multiple comparisons test, ns (non-significant), *p ≤ 0.05, **p ≤ 0.01, ***p ≤ 0.001)

To analyze the secretion efficiency of UniSePT SP for hIFNγ in comparison to industrially relevant human insulin (hINS) and human serum albumin (hALB) SP, we generated plasmid constructs in which the cDNA of hIFNγ was tagged with these three SPs at the N-terminal end of the protein (Fig. 5A). Comparative analysis showed that UniSePT SP had a significantly higher secretion efficiency with hIFNγ than industrially relevant hINS and hALB SPs (Fig. 5B). Furthermore, we examined the comparative ER localization of hIFNγ tagged with UniSePT, hINS, and hALB SP by co-transfecting with an ER localization signal-tagged mCherry construct. hIFNγ with no SP and with its native SP was used as a control for the experiment. High-content screening microscopy of co-transfected cells showed that UniSePT SP exhibited higher co-localization than industrially relevant hINS and hALB SPs (Fig. 5C). Transfection efficiency was analyzed by measuring EGFP expression via flow cytometry and copy number detection by qRT-PCR, which showed no significant changes across all experiments (Fig. S8-S9). To confirm the broad compatibility of UniSePT SP with other therapeutic proteins, we conducted an in silico study using the PrediSi tool on major commercially important recombinant proteins. We found that UniSePT showed minimal cleavage discrepancy and high secretion efficiency for most human therapeutic proteins compared to hALB-SP (Fig. 5D). SRP54 is a key component of the Signal Recognition Particle (SRP) and plays a pivotal role in the co-translational targeting of secretory and membrane proteins to the ER membrane. To visualize the interaction of UniSePT with SRP54, we performed docking analysis using ClusPro 2.0 with the ternary complex of the ribosome-nascent chain with SRP and Nascent polypeptide-associated complex (NAC) (PDB ID: 7QWQ). SRP54 recognizes and binds to the hydrophobic core of signal peptides, inducing conformational changes that are crucial for subsequent steps in protein targeting and translocation (Fig. S10). The specificity and selectivity of this interaction are essential for the efficient and accurate targeting of secretory and membrane proteins to the ER. Our docking analysis showed proper binding of UniSePT SP to SRP54 (Fig. 5E-F).

## Discussion

In recent years, most therapeutic proteins have been produced using mammalian cell-based expression systems, with a few produced in the milk of transgenic animal bioreactors [2, 4, 35]. A key limitation of these systems is the inconsistent yield and suboptimal biofunctionality of the expressed recombinant proteins [36–38]. The main reasons for these limitations are improper PTM in the ER and inefficient secretion through the ER-Golgi network [39]. One of the major bottlenecks in this process is the inefficient translocation of nascent proteins from the cytosol into the lumen of the endoplasmic reticulum (ER), a process that is heavily dependent on the presence and efficiency of the N-terminal signal peptide (SP) [16, 21, 40]. SPs facilitate the entry of proteins into the secretory pathway by engaging molecular chaperones that maintain nascent polypeptides in a translocation-competent state, thereby guiding proteins through the ER-Golgi network [14]. Efficient ER translocation is essential for proper post-translational modifications (PTMs), which ensure correct protein folding and bioactivity and influence secretion efficiency [41]. Multiple studies have shown that optimizing SPs is crucial for enhancing recombinant protein expression in mammalian systems. The literature also underscores that no SP is universally optimal for all protein-host combinations [15, 42]. Hence, the continued development of SP libraries, high-throughput screening strategies, and rational engineering approaches remains necessary; however, these are resource-intensive and delay the production timeline.

In the present study, we selected CSN2 and BLG-SP derived from different milk proteins of Indian river buffalo based on previous studies and our in silico analysis for further investigation. CSN2-SP from different sources has been successfully utilized to enhance the secretion of recombinant proteins, both in vitro and in vivo [27]. However, a study on the secretion of recombinant xylanase from *Pichia pastoris* showed that, although the bovine β-casein signal peptide could direct high-level secretion because of its strong hydrophobic core, most of them were trapped intracellularly [28]. In contrast to this, BLG-SP of goat origin was used to secrete recombinant human plasminogen activator in transgenic rabbits owing to its accurate cleavage properties [29]. To understand the role of the hydrophobic core in secretion efficiency, we chose the hIFNγ protein, which has the same predicted cleavage probability as CSN2 and BLG-SP. Similarly, we chose EGFP to study the effect of the cleavage site on secretion efficiency, as CSN2 introduces a + 5-amino-acid cleavage discrepancy in EGFP.

Based on our in vitro analysis, we found that CSN2-SP significantly increased the secretion of hIFNγ compared with native-SP, whereas it failed to secrete EGFP owing to its cleavage discrepancy. However, BLG-SP secreted significantly more EGFP than CSN2-SP, despite its relatively weaker hydrophobic core. We assumed that an incompatible cleavage site could account for the inefficiency of CSN2-SP tagged with the EGFP. This was confirmed by the inclusion of two amino acids (Arginine and Glutamine) in the CSN2-SP sequence (CSN2-RE), which restored EGFP secretion. Furthermore, removal of the c-region amino acids from BLG-SP (BLG-NoCS) resulted in a complete loss of secretion ability.

Our findings showed that neither CSN2-SP nor BLG-SP could universally drive robust secretion of hIFNγ or EGFP across different cell lines and protein types. CSN2-SP excelled for hIFNγ but lacked a versatile cleavage site, whereas BLG-SP was less efficient overall. We hypothesized that a hybrid signal peptide with an intact hydrophobic core of CSN2-SP and the C-region of BLG-SP would generate a robust broadly applicable Unique Secretory signal PepTide, i.e., UniSePT. Combining the structural features of differentially abled CSN2-SP and BLG-SP resulted in a potent SP with superior secretion efficiency for both secretory and non-secretory proteins, applicable across multiple mammalian cell lines of different tissue origin, including HEK293T, CHOK1, and primary goat mammary epithelial cells (GMECs). Notably, CHOK1 and HEK293T cells are widely used and are the major workhorses for industrial therapeutic protein production, whereas GMECs represent the target platform for transgenic animal bioreactors. As predicted, UniSePT significantly outperformed the parent SPs (CSN2 and BLG-SP) in terms of secretion capacity across all cell lines.

It is well known that conventional secretory SPs guide the co-translational insertion of the nascent polypeptide chain into the ER, followed by its translocation into the ER lumen, where it is further matured post-translationally and secreted out of the cells through the ER-Golgi pathway [16]. Hence, an efficient SP should robustly translocate exogenous proteins into the ER-Golgi network. To determine this, we co-transfected different SP-tagged EGFP constructs with ER-targeted mCherry constructs. Our study established that in terms of ER localization, UniSePT outperformed the parent CSN2-SP and BLG-SP.

Importantly, UniSePT retained high secretion efficiency across diverse mammalian expression platforms without requiring codon optimization, indicating its broad applicability for bioproduction across species. Codon optimization can significantly enhance the yield of recombinant proteins by substituting rare codons with more frequently used ones [43]. This is particularly important in heterologous expression systems, where the host organism may have a different codon usage bias than the source organism of the gene [43]. This requires an additional optimization step, which increases the production timeline. UniSePT can be used immediately without any further need for codon optimization.

Furthermore, the choice of signal peptide can influence the proper folding of the tagged protein and the occurrence of post-translational modifications, such as glycosylation. These modifications are critical for the biological activity and stability of several therapeutic proteins [44]. UniSePT was found to achieve higher secretion of the tagged therapeutic protein, hIFNγ, without compromising its biological activity.

Our comparative study also established that UniSePT significantly outperformed commonly used market-leading SPs, including those derived from human insulin (hINS) and human serum albumin (hALB), achieving expression levels up to 7-fold higher in CHOK1 cells. This could be attributed to the higher ER-targeting efficiency of UniSePT SP. These findings position UniSePT as a promising and broadly applicable SP for high-yield protein production applications. Previous studies have highlighted the protein-specific nature of SP performance, where an SP effective for one protein may be suboptimal for another; hence, efforts to identify or engineer universal SPs are ongoing. For example, in yeast, variants of the α-factor pre-proleader have evolved for broader utility. In mammalian systems, rationally designed SP panels have facilitated protein-specific optimization; however, truly universal SPs remain elusive [15]. In this context, in silico analyses using the SignalP 6.0 tool predicted the superiority of UniSePT in secreting diverse therapeutic proteins compared to other market-leading SP. These analyses suggest enhanced secretion efficiency, reduced N-terminal heterogeneity, and compatibility with nearly 80 therapeutic proteins [31].

Although these results substantially prove the efficiency of UniSePT, further experimental validation remains essential, given the complex interplay among the SP sequence, host cell machinery, and mature protein structure. The study was performed using a set of model proteins, primarily human interferon gamma (hIFNγ) and EGFP, at this initial stage of comparative analysis; however, to better elucidate the true potential of UniSePT, it is necessary to test it with a more diverse set of therapeutic proteins, particularly complex glycoproteins and multimeric proteins. In addition, as the study was limited to lab-scale culture conditions, it needs to be tested in large-scale culture environments, which are critical for industrial biomanufacturing. Furthermore, there is scope to elucidate the mechanistic basis of enhanced ER targeting and translocation, which was largely inferred in this study. This will further strengthen UniSePT’s impact on post-translational modifications (PTMs), including glycosylation patterns, folding kinetics, and protein stability.

Nevertheless, our findings demonstrate that UniSePT represents a significant step towards developing a broadly applicable SP that can improve yield while retaining the functionality of recombinant therapeutic proteins across a wide range of cellular platforms, without requiring additional sequence optimization.

## Conclusion

This study conclusively demonstrates the advantages of the newly developed engineered signal peptide, UniSePT, in significantly improving the yield of therapeutic proteins across mammalian systems without the need for further optimization of secretion efficiency or biofunctionality. This positions it as a valuable tool for generating higher protein titer, reducing production costs, and enhancing the overall efficiency of biotherapeutics manufacturing processes. The development and validation of UniSePT represent a significant step towards creating a broadly applicable signal peptide useful to a wide array of therapeutic proteins. Further research should focus on experimentally validating its effectiveness across a broader range of therapeutic proteins, including complex glycoproteins, and on optimizing its application in industrial bioproduction settings to fully realize its potential to revolutionize recombinant protein manufacturing.

## Supplementary Information

Below is the link to the electronic supplementary material.

Supplementary Material 1

## Data Availability

No datasets were generated or analysed during the current study.

## Abbreviations

BLG: β–Lactoglobulin
CHO: Chinese Hamster Ovary
CSN2: β–Casein
EGFP: Enhanced Green Fluorescent Protein
ER: Endoplasmic Reticulum
GMEC: Goat Mammary Epithelial Cells
hALB: Human Serum Albumin
HEK293T: Human Embryonic Kidney 293T cells
hIFNγ: Human Interferon Gamma
hINS: Human Insulin
HCS: High Content Screening
PTM: Post–Translational Modification
qPCR: Quantitative Polymerase Chain Reaction
SP: Signal Peptide
SRP: Signal Recognition Particle
SRP54: 54 kDa subunit of Signal Recognition Particle
