# Supplementary material for: UniSePT: an engineered broadly applicable Unique Secretory signal PepTide for high-yield biotherapeutic production in mammalian cell-based expression systems
Source: J Biol Eng. 2026 Jun 8;20:124. doi: 10.1186/s13036-026-00704-2 (PMC13471251; doi:10.1186/s13036-026-00704-2)
Supplement: Supplementary file 1 [file 13036_2026_704_MOESM1_ESM.docx]

**Title:** UniSePT: an engineered broadly applicable Unique Secretory signal PepTide for high-yield biotherapeutic production in mammalian cell-based expression systems.

**Authors:** Goutam Ulgekar^#,1^, Satarupa Dutta^#,1^, Ealisha Jain^1^, Meenakshi Mansukhani^1^, Harshada Thawari^1^, Subeer S. Majumdar^1,2^, Nirmalya Ganguli^1,*^

1. Gene and Protein Engineering Laboratory, National Institute of Animal Biotechnology, Hyderabad, Telangana, India.
2. Present Address: Gujarat Biotechnology University, Gandhinagar, Gujarat.

**#**Contributed equally to this study

*Corresponding Author: Nirmalya Ganguli, PhD.

Gene and Protein Engineering Laboratory,

National Institute of Animal Biotechnology, Hyderabad,

Telangana, India.

E. Mail: [nganguli@niab.org.in](mailto:nganguli@niab.org.in)

**Supplementary Figure 1**

**pCMV-hIFNγ-IRES2-EGFP**

**A.**

**B.**

**Figure. S1**: A. Construct map of pCMV-hIFNγ-IRES2-EGFP B. Subcloning of SPs showing N-region(Red), H-region (green) and C-region (yellow) at N-terminus of hIFNγ.

**Supplementary Figure 2**

**A.**

**B.**

**Figure. S2:** Quantification of hIFNγ in spent media of A. HEK293T and B. Goat mammary epithelial cells (GMEC) transfected with plasmid construct expressing hIFNγ with Native, CSN2 and BLG SP*.* The plasmid construct having no signal sequences attached to the cDNA of the hIFNγ (NoSP-hIFNγ) at the N terminal end was used as control. The data indicates mean and SEM, n= 3 independent experiments. Data were analyzed by two-tailed t test, ns (nonsignificant), *p ≤ 0.05, **p ≤ 0.01, ***p ≤ 0.001).

**Supplementary Figure 3**

**A.**

**B.**

**Figure. S3**: A. Construct map of pCMV-EGFP B. Subcloning of SPs showing N-region(Red), H-region (green), C-region (Yellow) at N-terminus of EGFP.

**Supplementary Figure 4**

**A.**

**B.**

**C.**

**Figure. S4:** Flow cytometric analysis of A.HEK293T, B. CHO K1, C. Goat mammary epithelial cells transfected with plasmid construct (pCMV-hIFNγ-IRES2-EGFP) expressing signal peptides (hIFNγ, Csn2 and Blg). The data indicates mean and SEM, n= 3 independent experiments. Data were analysed by ordinary one‐way ANOVA (Tukey's multiple comparisons test), ns (nonsignificant), *p ≤ 0.05, **p ≤ 0.01, ***p ≤ 0.001).

**Supplementary figure 5**

**Figure. S5:** Flourescent microscopic images of CHO K1 cells transfected with plasmid construct (pCMV-EGFP) having signal peptides (CSN2, CSN2+RE, BLG, BLG(NoCS)) and UniSePT signal peptide.

**Supplementary Figure 6**

**Figure. S6:** Flow cytometric analysis of CHO K1 transfected with plasmid construct (pCMV-hIFNγ-IRES2-EGFP) having signal peptides (CSN2, CSN2+RE, BLG, BLG(NoCS)) and UniSePT signal peptide. The data indicates mean and SEM, n= 3 independent experiments. Data were analysed by ordinary one‐way ANOVA (Tukey's multiple comparisons test), ns (nonsignificant), *p ≤ 0.05, **p ≤ 0.01, ***p ≤ 0.001).

**Supplementary Figure 7**

**Figure. S7:** Copy number analysis of CHO K1 cells transfected with plasmid constructs having signal peptides (CSN2, CSN2+RE, BLG, BLG(NoCS)) and UniSePT signal peptide. The data indicates mean and SEM, n= 3 independent experiments. Data were analyzed by ordinary one‐way ANOVA (Tukey's multiple comparisons test), ns (nonsignificant), *p ≤ 0.05, **p ≤ 0.01, ***p ≤ 0.001).

**Supplementary Figure 8**

**A.**

**B.**

**C.**

**Figure. S8:** Flow cytometric analysis of A.HEK293T, B. CHO K1, C. Goat mammary epithelial cells transfected with plasmid construct (pCMV-hIFNγ-IRES2-EGFP) having signal peptides (CSN2, CSN2+RE, BLG, BLG(NoCS)and UniSePT signal peptide) and with plasmid constructs having milk signal peptides and other robust signal peptides i.e. Human insulin (HINS) and human serum albumin (HSA). The data indicates mean and SEM, n= 3 independent experiments. Data were analysed by ordinary one‐way ANOVA (Tukey's multiple comparisons test), ns (nonsignificant), *p ≤ 0.05, **p ≤ 0.01, ***p ≤ 0.001).

**Supplementary Figure 9**

**Figure. S9:** Copy number analysis of CHO K1 transfected with plasmid construct (pCMV-hIFNγ-IRES2-EGFP) having signal peptides (CSN2, CSN2+RE, BLG, BLG(NoCS)and UniSePT signal peptide) and with plasmid constructs having milk signal peptides and other robust signal peptides i.e. Human insulin (hINS) and human serum albumin (hALB). The data indicates mean and SEM, n= 3 independent experiments. Data were analyzed by ordinary one‐way ANOVA (Tukey's multiple comparisons test), ns (nonsignificant), *p ≤ 0.05, **p ≤ 0.01, ***p ≤ 0.001).

**Supplementary Figure 10**

**Figure. S10:** Ternary complex of ribosome nascent chain with SRP and NAC (PDB ID: 7QWQ)
